# A decade of *Streptococcus pneumoniae* bloodstream infections: a single-center retrospective analysis

**DOI:** 10.1017/ash.2026.10369

**Published:** 2026-04-10

**Authors:** Saied Ali, Karen Burns, Binu Dinesh, Helene McDermott, Sinead O’Donnell, Fidelma Fitzpatrick, Ciara O’Connor

**Affiliations:** 1 Microbiology, RCSI University of Medicine and Health Sciences: RCSI Dublin, Ireland; 2 Microbiology, Beaumont Hospital, Ireland

## Abstract

A ten-year retrospective review of 176 pneumococcal bloodstream infections found broad-spectrum antimicrobial use despite high penicillin susceptibility. Complications occurred in 15%, and ICU admission in 10%. Only 22% had pneumococcal vaccination recommended at discharge. Findings reveal missed opportunities for Antimicrobial Stewardship and preventative care in invasive pneumococcal disease management.

## Introduction

*Streptococcus pneumoniae* remains a leading cause of global morbidity and mortality, with clinical manifestations ranging from mild otitis media to life-threatening invasive pneumococcal disease (IPD), encompassing pneumonia, bloodstream infection (BSI), meningitis and septic shock.^1^ Vulnerable populations, such as infants, older adults and immunocompromised individuals are particularly affected, with annual incidence in adults ≥65 years in Europe of 18–19 cases per 100,000.^2^ In Ireland, IPD persists as a substantial public health burden. According to the Irish Health Protection Surveillance Centre (HPSC), pneumococcal BSI and meningitis are among the commonest vaccine-preventable causes of morbidity. In 2023, there were 433 confirmed cases in Ireland, corresponding to an incidence rate of 8.4 per 100,000 population, an increase from 7.3 in 2022.^3^

The respiratory tract remains the primary entry point for *S. pneumoniae*, with pneumonia as the most common clinical presentation. Severe infection frequently progresses to BSI, requiring critical care admission, with complications such as empyema, lung abscess and central nervous system (CNS) involvement resulting in considerable mortality and life-changing morbidity among IPD survivors.^1^

Importantly, IPD is largely preventable through vaccination. Evolution of conjugate (e.g., PCV7 to PCV13 to PCV20) and polysaccharide vaccines (e.g., PPV23) target multiple invasive serotypes and have reduced IPD in both pediatric and adult populations. In Ireland, PCV is part of the routine childhood immunization program, with PPV23 recommended for high-risk groups and adults ≥65 years.^3,4^

Gaps remain with regard to opportunities to de-escalate from broad to narrow-spectrum antimicrobial therapy upon microbiological confirmation of IPD, and missed opportunities for prevention through vaccination. This study analyses a decade of pneumococcal BSIs to evaluate clinical practices, with the aim of identifying opportunities to improve treatment and prevention.

## Methodology

A retrospective review of all laboratory-confirmed *S. pneumoniae* BSI cases, hereafter termed pneumococcal BSIs, in one tertiary hospital [850-beds] between January 1^st^ 2014 and December 31^st^ 2023 was undertaken. Cases were identified through the laboratory information system, from which patient demographics, antimicrobial susceptibility profiles and serotype (when available) were extracted.

Healthcare records were reviewed to ascertain recognized risk factors for IPD, clinical complications, intensive care unit (ICU) admission, definitive antimicrobial therapy, total duration of antimicrobial treatment, and intravenous (IV) to oral (PO) switch therapy (IVOST), where applicable. Documentation of pneumococcal vaccination recommendation at discharge was also recorded. To further characterize vaccination status, the patient’s family physician was contacted to ascertain pneumococcal vaccination history prior to and following hospitalization.

Descriptive statistics were used to summarize clinical characteristics, antimicrobial prescribing, serotype distribution and trends in preventive practices. Analysis was performed using Microsoft Excel (Microsoft Corp., Redmond, WA, USA).

## Results

In total, 176 pneumococcal BSIs were identified between 2014 and 2023. Annually, cases were highest in 2018 (n = 28), 2017 (n = 26) and 2023 (n = 25), with the lowest incidence during the first two years of the Coronavirus Disease 2019 (COVID-19) pandemic—2020 (n = 9) and 2021 (n = 7) (Table 1).

**Table 1.** Annual summary of *Streptococcus pneumoniae* bloodstream infections (2014–2023): antimicrobial resistance, clinical complications and de-escalation practices

Year	*Streptococcus pneumoniae* BSIs, n	Antimicrobial non-susceptible isolates, n			Complications, n	ICU admission, n	De-escalated to narrower spectrum antimicrobial agent, n
		Penicillin	Erythromycin	Cefotaxime			
2014	14	1	1	0	3	1	5
2015	18	5	2	0	5	2	2
2016	19	3	3	1	4	3	4
2017	26	5	2	2	4	3	2
2018	28	2	2	0	1	5	4
2019	14	1	1	1	0	1	5
2020	9	0	0	0	2	2	2
2021	7	1	0	0	2	0	2
2022	16	3	3	0	2	0	6
2023	25	6	4	0	3	1	5

BSI, bloodstream infections, ICU, intensive care unit.

The median patient age was 69.5 years (IQR: 58.8–79.3; range: 17–94), with 56% of BSIs (n = 99) in patients ≥65 years. Females accounted for 53% (n = 93 cases). Among documented risk factors for IPD ascertained from healthcare records, diabetes mellitus was documented in 16% (n = 28), chronic heart disease in 29% (n = 51), chronic pulmonary disease in 11% (n = 19), chronic liver disease in 4% (n = 7), and chronic kidney disease in 26% (n = 46). Nine patients (5%) had ≥2 of these risk factors. Prior pneumococcal vaccination was reported in 55 patients (31%). Data on the type of vaccine was unavailable.

The presumed BSI source was respiratory in 138 cases (78%), central nervous system in five (3%), abdominal in two (1%), and unknown for the remaining cases.

Complications included; empyema (n = 13; 7%), lung abscess (n = 8; 5%) and meningitis (n = 5; 3%). Admission to ICU occurred in 18 cases (10%). Overall, recorded in-hospital mortality was 6% (n = 10), either directly or indirectly attributable to IPD.

A high proportion of isolates were susceptible to penicillin (n = 149; 85%) and cefotaxime (n = 172; 98%). Non-susceptibility to erythromycin, as a proxy for macrolide resistance, was observed in 21 isolates (12%). Among 149 penicillin-susceptible isolates, only 37 (25%) were de-escalated to a narrow-spectrum *β*-lactam (e.g., amoxicillin or penicillin).

Overall, the median total duration of antimicrobial therapy was 10 days (IQR 10–14). Median IV duration was 3.5 days (IQR 3–4), and among those undergoing IVOST, the median PO duration was 7 days (IQR 7–7). IV therapy alone was used in 41 cases (23%), whereas 135 (77%) underwent IVOST. Nine patients completed their IV course via outpatient parenteral antimicrobial therapy (OPAT).

Empyema (n = 13) and lung abscess (n = 8) were treated for a median of 42 days, with IVOST undertaken in eight and four of those cases, respectively. Meningitis (n = 5) was managed exclusively with IV antimicrobials for a median of 14 days. Other cases requiring ICU admission (n = 18) received a median of 14 days of therapy, with five undergoing IVOST. Among non-ICU cases without focal complications (n = 132), the median treatment duration was 10 days, and 128 (97%) underwent IVOST to complete treatment.

**Figure 1.**
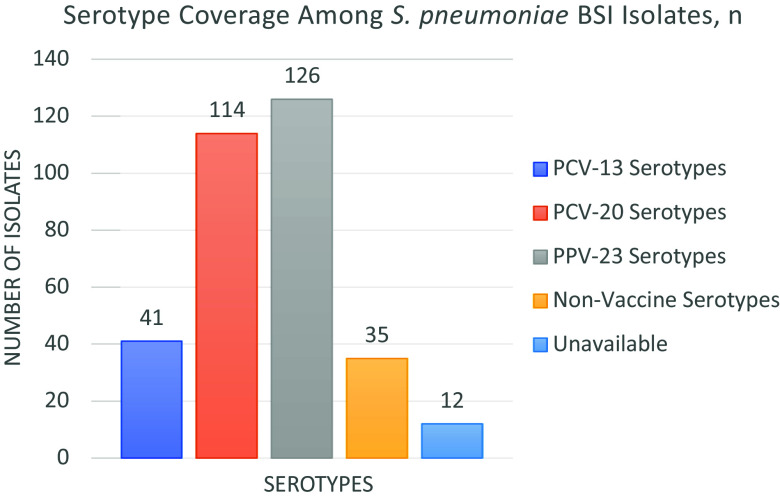
Distribution of *Streptococcus pneumoniae* Serotypes by Vaccine Coverage (2014–2023). BSI, bloodstream infections.

Of the 176 cases, serotype analysis was available for 164 (93%), with the most frequently identified serotypes being 8 (n = 31), 19A (n = 15), 22F (n = 14), 12F (n = 11) and 3 (n = 9). Serotypes included in PCV13 accounted for 25% (n = 41) of cases. Serotypes included in PCV20 accounted for 70% (n = 114), representing an absolute increase of 45% compared with PCV13. PPV23-covered serotypes comprised 77% (n = 126) of isolates (Figure 1). Non-vaccine serotypes, including 15A, 23A, 23B, 35B, 6C and 7C, were identified in 21% (n = 35).

Despite having laboratory-confirmed pneumococcal BSI, just 38 patients (n = 22%) had a recommendation for pneumococcal vaccination documented at discharge. Of these, only 26 were subsequently vaccinated upon confirmation with their family physician.

## Discussion

This ten-year review of pneumococcal BSIs identified opportunities for improvement in clinical practice. With regard to Antimicrobial Stewardship (AMS), it was uncommon for patients to be de-escalated from empiric broad spectrum empiric agents to narrow spectrum penicillins, despite availability of antimicrobial susceptibility results to support such decisions. This limited de-escalation to narrow-spectrum *β*-lactams suggests that therapeutic decisions were often guided by routine clinical practice or clinician preference for broad-spectrum coverage rather than tailored to microbiological confirmed data.^5^ Targeted prescriber education, coupled with structured AMS support may facilitate more appropriate de-escalation of therapy.^5^ Additionally, embedding automatic AMS review of positive blood cultures, incorporating electronic prescribing prompts at the time of result verification, and standardizing bacteremia management pathways may enhance microbiology-directed optimization of therapy and strengthen AMS impact.^6^ On a more positive note, the proportion of IVOST was high, with over three-quarters of patients transitioned to PO therapy after a short median IV duration of 3.5 days.

The observed overall mortality rate of 6% was lower than historical estimates of IPD mortality among older adults, which have typically ranged between 10%–20% in international cohorts.^7^ This may reflect improvements in early clinical recognition, timely commencement of antimicrobial therapy, and advances in supportive care. However, ICU admission in 10% of cases underscores the clinical severity associated with IPD.

The decline in pneumococcal BSIs during 2020–2021 coincided with the COVID-19 pandemic, likely reflecting the indirect effects of non-pharmaceutical interventions, including reduced social contact, widespread use of face masks or coverings and mobility restrictions, which were associated with marked declines in respiratory pathogen transmission.^8^ Contemporaneous national laboratory surveillance data in Ireland also recorded a reduction in typed isolates from patients with IPD from 374 (2019) to 181 (2020) and 160 (2021), followed by a resurgence to 379 (2023).^3^ In addition, changes in healthcare utilization during the pandemic, including reduced emergency department attendance for non-COVID respiratory illness and modified hospital admission practices, may have influenced case ascertainment.^9^ Comparable reductions in invasive bacterial disease, including *S. pneumoniae*, were reported across multiple countries during periods of strict COVID-19 control measures;^8^ however, the subsequent return to prepandemic case numbers underscores that these reductions were transient and mainly reflective of behavioral and public health interventions rather than a sustained change in pneumococcal epidemiology.

Consequently, prevention of IPD remains a public health priority, particularly among older adults who bear the highest disease burden in Ireland, with an incidence rate of 24.7 per 100,000 in ≥65 years in 2023.^3^ PCV and PPV are available as part of national immunization strategies. Despite this, prior pneumococcal vaccination was documented in only 31% of patients in our cohort. Furthermore, despite pneumococcal BSI being a clear indication for immunization, a vaccination recommendation at discharge was recorded in just 22% of cases, and only 26 of 38 recommendations were subsequently actioned. These findings highlight missed opportunities for secondary prevention.

Ireland introduced PCV7 into the childhood immunization schedule in 2008, replaced by PCV13 in 2010, resulting in substantial reductions in vaccine-type IPD and indirect protection in older age groups through herd immunity.^10^ However, uptake of three PCV doses by 24-months has declined from 92.5% in 2015 to approximately 82% nationally in recent years.^10,11^ The median age in our cohort was 69.5 years, indicating that most patients would not have benefited from routine childhood PCV immunization and instead relied on adult vaccination strategies. PPV23 is recommended in Ireland for adults aged ≥65 years and for younger adults with defined risk factors. Since 2015, a sequential strategy of PCV13 followed by PPV23 has been recommended for adults with immunocompromising conditions or significant comorbidity, reflecting efforts to broaden protection in high-risk groups.^10^ However, adult uptake has historically been low, 27%–36%, and adult vaccination often depends on patient health-seeking behavior, opportunistic primary care engagement, and clinician recommendation.^11^ Vaccine hesitancy, competing health priorities, and lack of systematic recall mechanisms may further contribute to suboptimal uptake.^12^

The high prevalence of chronic diseases (heart, kidney and pulmonary) and diabetes mellitus in this cohort suggests that many patients would likely have been vaccine candidates prior to their IPD case. The low rates of documented vaccination and discharge recommendation suggest a need for system-level interventions. Embedding vaccination assessment into standardized discharge protocols, incorporating electronic prompts, and strengthening communication with family physicians may improve vaccine uptake. Education of both healthcare professionals and patients regarding the importance of pneumococcal vaccination following IPD is also warranted.^13^

Serotype distribution in this study further informs vaccine strategy. While only 25% of cases were attributable to PCV13 serotypes, 70% would be covered by PCV20, and 77% by PPV23. These findings support consideration of higher-valency conjugate vaccines in immunization programs and reinforce the importance of continued surveillance to guide national vaccine policy.^10,14^ Given Ireland’s aging population and the burden of comorbidity observed, systematic assessment of pneumococcal vaccination status in community-based care settings, as well as during hospital encounters should be prioritized.

This study provides information about pneumococcal BSI over a ten-year period, but there are several limitations. Most BSI (78%) were attributed to a respiratory source, indicative of IPD most commonly arising in the context of pneumonia. The focus of this study was laboratory-confirmed BSI as a robust and microbiologically-defined end point. Non-bacteremic pneumococcal infections, including community-acquired pneumonia were not captured and likely represent a substantial additional burden of disease. Nonetheless, the observed patterns in serotype distribution, vaccination gaps, and antimicrobial management in IPD have clear implications for the broader clinical spectrum of pneumococcal infection. The retrospective design inherently relies on the completeness and accuracy of existing medical records and laboratory data, which may have led to under-reporting of complications, vaccination documentation, notably oral advice provided to patients regarding vaccination was not captured, or treatment decisions. Secondly, pneumococcal serotyping was not available for 7% of cases, potentially limiting the accuracy of vaccine coverage estimates. Thirdly, the absence of broader hospital denominator data precludes assessment of testing rates or prescribing behaviors across all patients with suspected pneumococcal disease. In addition, HIV serostatus and documented allergy status were not routinely available in the data set; both may have influenced clinical decisions around suitability for de-escalation from a cephalosporin to a penicillin for example. Furthermore, information on certain recognized risk factors for IPD—including alcohol misuse, asplenia or hyposplenism, and other forms of immunosuppression—were not consistently available, and the burden of comorbidity in this cohort is therefore likely underestimated. Finally, data from a single institution may limit generalizability, although findings are consistent with broader national trends and highlight systemic gaps in diagnostic and preventive practices.

## Conclusion

This ten-year review of pneumococcal BSI identified both strengths and gaps in clinical practice. While early IVOST was consistently implemented, with short median IV durations, opportunities remain to improve de-escalation from broad- to narrow-spectrum antimicrobials upon availability of antimicrobial susceptibility results. In parallel, low rates of prior vaccination and limited discharge recommendations highlight missed opportunities for secondary prevention. Addressing these gaps through targeted AMS interventions and structured, system-level vaccination strategies will be essential to optimize care and reduce recurrent IPD.

## Data Availability

All data generated or analyzed during this study are included in this published article.
